# Differential Neutralization Profiles of 17DD Vaccinated Population to 17D-204 and 17DD Vaccine Strains

**DOI:** 10.3390/vaccines12121311

**Published:** 2024-11-23

**Authors:** Ana C. B. Terzian, Sasha R. Azar, Cassia F. Estofolete, Mauricio L. Nogueira, Nikos Vasilakis

**Affiliations:** 1 Laboratório de Imunologia Celular e Molecular, Instituto René Rachou, Fundação Osvaldo Cruz, Belo Horizonte 30190-009, MG, Brazil; anacarolinaterzian@gmail.com; 2 Center for Tissue Engineering, Department of Surgery, Houston Methodist Research Institute, Houston Methodist Hospital, Houston, TX 77030-2703, USA; srazar@houstonmethodist.org; 3 Department of Pathology, University of Texas Medical Branch, Galveston, TX 77555-0609, USA; 4 Laboratório de Pesquisas em Virologia, Departamento de Doenças Dermatológicas, Infecciosas e Parasitárias, Faculdade de Medicina de São José do Rio Preto, São José do Rio Preto 15090-000, SP, Brazil; cassia.estofolete@edu.famerp.br; 5 Hospital de Base, Fundação Faculdade Regional de Medicina de São José do Rio Preto, São José do Rio Preto 15090-000, SP, Brazil; 6 Center for Vector-Borne and Zoonotic Diseases, University of Texas Medical Branch, Galveston, TX 77555-0609, USA; 7 Institute for Human Infection and Immunity, University of Texas Medical Branch, Galveston, TX 77555-0610, USA

**Keywords:** YFV, vaccine, immune response, neutralizing antibodies

## Abstract

**Background/Objectives**: Yellow fever virus (YFV) (*Flaviviridae*, *Orthoflavivirus*) is the etiologic agent of yellow fever (YF), a vector-borne disease with significant morbidity and mortality across the tropics and neotropics, despite having a highly efficacious and safe vaccine (17D). Vaccination provides lifelong protection from YF disease mediated by humoral immunity. There are several versions of the original 17D vaccine: 17D-204 (marketed in the USA as YF-VAX, in France as Stamaril, and in China as Tiantan-V), 17D-213 (Russian Federation), and 17DD (by FIOCRUZ in Brazil). Vaccines produced in the US, France, Senegal, China, and Russia represent 17D-204-derived strains, whereas the Brazilian 17DD has a unique passage/attenuation history from 17D-204-derived strains. Their functional differences in the neutralization profiles are not known. **Methods**: The Plaque Reduction Neutralization Test (PRNT) was used to determine the neutralization profiles of sera from 209 patients that were previously vaccinated with the 17DD strain against both 17D-204 and 17DD. **Results**: Sera exhibited significantly more efficient neutralization of 17DD (mean reciprocal PRNT_50_ 183, PRNT_80_ 86, median reciprocal PRNT_50_ 80, and PRNT_80_ 40) compared to 17D-204 (mean reciprocal PRNT_50_ 91, PRNT_80_ 33, median reciprocal PRNT_50_ 40, and PRNT_80_ 10). **Conclusions**: Our data indicate antigenic differences between 17D and 17DD vaccines.

## 1. Introduction

Mosquito-borne orthoflaviviruses include several pathogens of global importance, particularly dengue (DENV), Zika (ZIKV), yellow fever (YFV), Saint Louis Encephalitis (SLEV), Japanese encephalitis virus (JEV), and West Nile (WNV) viruses [1]. Orthoflavivirus infection induces lifelong immunity, mediated by antibodies and T cells [1,2]. To date, there are licensed vaccines against YFV, TBEV, DENV, and JEV [3,4,5]. Among these, the YFV-17D vaccine is considered one of the most successful vaccines, demonstrating safety and efficacy from a single dose, with almost 100% seroconversion 10 days post-vaccination, robust production of neutralizing antibodies, and lifelong immunity [6,7,8,9]. There are several attenuated versions of the original 17D vaccine: 17D-204 (marketed in the USA as YF-VAX, in France as Stamaril, and in China as Tiantan-V); 17D-213 (Russian Federation); and 17DD (by FIOCRUZ in Brazil), which is only licensed in Brazil.

Yellow fever mass vaccination has proven to be effective [10], and today the worldwide control of the disease is achieved through the use of live-attenuated vaccines of the sub-lineages 17DD, 17D-204, and 17D-213, a sub-lineage derived from 17D-204 [11,12]. All sub-lineages are derived through serial passaging from the parental Asibi strain isolated from a febrile patient in Ghana in 1927 [2,13,14] to produce seed lots of 17D. The genomic differences between the 17D sub-lineages and the parental Asibi are 48 nucleotides (four in the 3′UTR region) and 20 amino acids [11,12]. These mutations are considered the molecular basis of attenuation for the vaccine strains [11].

YF remains to this day a significant threat in many regions of Latin America and Africa; thus, our study evaluating the serological response to different YF vaccine strains is crucial for understanding their immunogenic efficacy. Importantly, assessing the serological response is essential to identify potential limitations in the protection offered by each vaccine strain, optimize subsequent immunization strategies to obtain better vaccine coverage, and, lastly, to inform public health policies for potential adjustments in vaccination recommendations.

The literature documents that induced immune responses can vary significantly between different vaccine strains due to differences in vaccine preparation, genetic variability of the vaccine virus, and individual characteristics of vaccine recipients [15,16], although the homology between the 17DD and 17D-204 vaccines is 99% [10]. Thus, we wanted to assess whether there would be a difference in the neutralizing response in sera obtained from individuals residing in the hyper-endemic city of São José do Rio Preto, Sao Paulo State, Brazil.

## 2. Materials and Methods

### 2.1. Study Site

The city of São José do Rio Preto (SJdRP) is located in the northwestern region of the State of São Paulo, Brazil, with an estimated population of 450,657 inhabitants. SJdRP has a tropical climate with a mean annual temperature of 25 °C and mean rainfall of 1410 mm, concentrated mostly in the summer months. According to the Brazilian Institute of Geography and Statistics (Instituto Brasileiro de Geografia e Estatística—IBGE), the city has development indices comparable to those of developed countries, and its economy encompasses industry, services, commerce, and agrobusiness. The city has experienced hyper-endemic circulation of DENV for over 20 years, with detection of all four serotypes. Currently, SJdRP is heavily infested with *Ae. aegypti*, *Ae. albopictus*, and *Culex* sp., and the city has become a hotspot of arboviruses surveillance programs due to active circulation of DENV, YFV, ZIKV, chikungunya (CHIKV), SLEV, and possibly others (e.g., Mayaro). SJdRP is included in the YF vaccine recommendation area according to the Brazilian Ministry of Health and has a high vaccination coverage, which, at the time of our study, stood at 69.28% [17].

From October 2015 to early 2016, a 1500-person prospective dengue and Zika cohort, ranging in age from 10 to 91 years, was conducted in Vila Toninho neighborhood, a representative area of the city. The site was selected due to the close proximity of health-care facilities. The neighborhood is surrounded by vegetation with an estimated population of 8700 residents, aged 01–59 years of age, and 10,815 inhabitants in total.

### 2.2. Sample Collection

We analyzed the YF vaccine responses from convenience samples selected from two different cohorts: (i) acute febrile illness patients (named symptomatic patients) and (ii) Vila Toninho prospective dengue cohort (named cohort group). After that, the samples were paired by sex and age into each group.

(i) *Symptomatic patients*. A total of 46 blood samples were collected from patients presenting with acute febrile Zika-confirmed symptoms that were treated by the Public Health Authority, in the city of São José do Rio Preto (SJdRP). The permission to use these samples is part of an ongoing arbovirus surveillance program approved by the local research ethics committee (EC No. 02078812.8.0000.5415, approved on 3 July 2012) and did not require any written or oral informed consent.

(ii) *Cohort group*. A total of 163 samples were collected from patients enrolled in a prospective dengue study enrolled at the Family Health Program Unit (FHPU) in Vila Toninho, a known area by *Aedes* spp. infestation. A total of 1500 permanent residents in the area were eligible and aged ≥ 10 years. At the time of sampling, none of them was presenting clinical symptoms compatible with arboviral infection. The cohort study was approved by the local research ethics committee (EC number 32993014.0.0000.5415, approved on 9 September 2014), and all subjects provided written informed consent.

### 2.3. Virus

Yellow fever vaccine strain 17D-204 was provided by Dr. Alan D.T. Barrett (University of Texas Medical Branch) and was passaged 3 times on Vero cells (clone CCL-81, ATCC, Manassas, VA, USA) to produce a large-scale stock of sufficient titer. Yellow fever vaccine strain 17DD was acquired from WRCEVA (TPV-5206) (TX, USA) as a lyophilized stock and was reconstituted in 0.5mL of sterile molecular biology-grade water. Reconstituted 17DD underwent a total of 2 passages on Vero cell to produce a large-scale stock of sufficient titer.

### 2.4. Plaque Reduction Neutralization Test

Sera were assayed to determine the specific neutralization antibody titers, as described previously [18,19]. Briefly, the PRNT assay was performed in 24-well plates of 90% confluent Vero cells, using a fixed challenge virus inoculum (approximately 60 focus-forming units) against varying serum dilutions (1:20–1:640). Following 5 days of incubation at 37 °C in a 5% CO_2_ incubator, the plates were then fixed with cold 1:1 methanol/acetone, and foci were stained immunologically with mouse anti-YFV ascites fluid (1:2000), as previously described [18,19]. The PRNT titers were scored as reciprocal of the highest dilution of serum that inhibited 50% or 80% of plaques (PRNT_50_ or PRNT_80_). Samples scored as PRNT_50_ < 20 or the more stringent cutoff PRNT_80_ < 20 were considered negative. During statistical analysis (detailed below), values below the limit of detection were assigned the reciprocal PRNT value of ½ of the assay limit of detection.

### 2.5. Statistical Analysis

Median reciprocal PRNT titers between challenge with 17D-204 and 17DD were compared using two-tailed pair-wise Mann–Whitney/Wilcoxon rank-sum test. Statistical analyses were performed using GraphPad Prism version 10.2.3 software (GraphPad Software, San Diego, CA, USA).

## 3. Results

In total, 209 serum samples, comprising 46 presenting with Zika fever and 163 non-symptomatic cohort controls, were evaluated for differences in neutralization response to YF vaccines strains 17D-204 and 17DD. Of the 209 serum samples at PRNT_50_, 26/209 (12.4%) and 46/209 (22%) were tested seronegative against 17DD and 17D-204, respectively (Raw Data). Of the 26 samples found to be seronegative by 17DD challenge, only two (samples 1358 and 1444) were positive by 17D-204, albeit both were within two plaques of reaching the PRNT_50_ cutoff value for 17DD.

**Figure 1:**
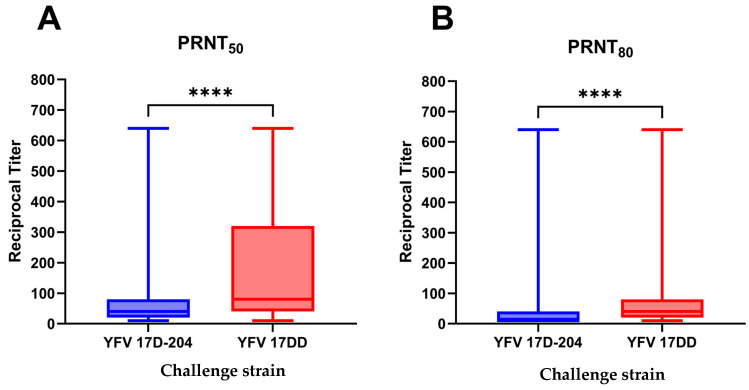
Reciprocal Plaque Reduction Neutralization Test (PRNT) titers of the study population. Reciprocal neutralization titers of the 209 serum samples against either YFV 17D-204 or 17DD. (**A**) Representation of 50% neutralization titer against designated vaccine strain. (**B**) Representation of 50% neutralization titer against designated vaccine strain. Bold lines within the boxes represent median values. Between-group comparisons were made using the nonparametric Mann–Whitney test to detect differences in median values. **** *p* < 0.0001.

Our analysis of the sera by the PRNT_50_ cutoff values yielded reciprocal neutralization titers ranging from below the lower limit of detection (PRNT_50_ < 20) to the upper limit of the assay (PRNT_50_ < 640) for both 17D-204 and 17DD. For 17D-204, the mean reciprocal titer was 90.86 with a standard error of 8.72, with titers of 20 and 80 serving as the 25 and 75% quartiles. 17DD challenge resulted in a mean reciprocal titer of 183.1 with a 14.19 standard error of the mean (SEM), and titers of 40 and 320 serving as the 25 and 75% quartiles. The median reciprocal titer of 17D-204 was 40, significantly lower than that of the median reciprocal titer of 17DD, i.e., 80 (95%CI 80–160; *p* < 0.0001; Figure 1A).

Our analysis of the sera by the PRNT_80_ cutoff yielded reciprocal neutralization titers ranging from below the lower limit of detection (PRNT_80_ < 20) to the upper limit of the assay (PRNT_80_ < 640) for both 17D-204 and 17DD challenge. For 17D-204, the mean reciprocal titer was 33.11 with a standard error of 4.15, with titers of 10 and 40 serving as the 25 and 75% quartiles. 17DD challenge resulted in a mean reciprocal titer of 86.17 with a 9.23 SEM, and with titers of 20 and 80 serving as the 25 and 75% quartiles. The median reciprocal titer of 17D-204 was below the limit of detection (LoD) of the assay (set to ½ LoD, or 10 for statistical testing), significantly lower than that of the median reciprocal titer of 17DD, i.e., 40 (*p* < 0.0001; Figure 1B).

## 4. Discussion

This study provides compelling evidence of the differential neutralization profiles between the 17DD and 17D-204 YF vaccine strains in the general population. The results show higher titers of neutralizing antibodies to homologous 17DD strain compared to the heterologous 17D-204 strain. This difference is seen in both PRNT_50_ and PRNT_80_ titers, where the 17DD strain consistently outperformed 17D-204.

Despite the remarkable success of the YFV-17D vaccine, some controversy exists around the immune response elicited by its different sub-lineages, and questions remain regarding whether this translates to equivalent immunogenicity and protection levels. Previous research has indicated that even minor genomic variations can influence vaccine efficacy and immune responses [12]. YFV shares significant antigenic similarities with other members of the genus *Orthoflavivirus*, such as DENV and ZIKV, leading to the generation of cross-reactive antibodies in individuals who have been exposed to either of the viruses [20]. These cross-reactive immune responses can complicate serological testing, often yielding false positives that obscure the differentiation between past dengue and yellow fever infections [21]. Moreover, this cross-reactivity has implications for vaccine efficacy and safety. Dengvaxia, the first licensed dengue vaccine, showed that individuals without prior dengue exposure could experience severe dengue disease upon subsequent heterologous infection due to antibody-dependent enhancement (ADE), where cross-reactive, non-neutralizing antibodies exacerbate the infection rather than prevent it [22]. Additionally, vaccination against YF in dengue-endemic regions has shown that cross-reactive antibodies may amplify immune responses during subsequent dengue infections, complicating diagnoses and potentially worsening the disease [16]. Japanese encephalitis virus (JEV) vaccination can also induce cross-reactive antibodies that bind to DENV, leading to false positives in serological tests and complicating vaccine decisions in dengue-endemic areas [23].

A double-blind, randomized, placebo-controlled trial, also performed in Brazil, observed a similar seroconversion rates of around 98% among previous seronegative participants and who were vaccinated with 17D-204 and 17-DD and tested after 30 days from vaccination) [24]. In a prospective cohort study on seroconversion of the 17-DD yellow fever vaccine, 98.3% of individuals exhibited reactive anti-YF IgG 30 days after immunization, while 100% tested positive via the PRNT_50_ method. The cohort also included a retrospective evaluation of individuals vaccinated 5 or 10 years earlier, where anti-YFV IgG rates of 60% and 55% were observed, respectively, while 100% of individuals had detectable antibodies through PRNT at the same time intervals [25]. In another study evaluating the 17D-204 vaccine, a 100% neutralizing antibody rate was also observed using PRNT_90_ two weeks post-vaccination (or, in which cases with seroconversion did not occur after one dose, seroconversion was assessed after a second dose) [26].

The previously mentioned data highlight high rates of immune response in the initial weeks following vaccination, with a gradual decline over the years, particularly concerning the 17D-204 vaccine. However, while these observations offer a basis for understanding the long-term efficacy of these vaccines, they do not thoroughly address the differences in neutralization profiles between the two vaccine strains.

Our findings highlight the antigenic differences between these two vaccine sub-lineages despite their high genomic homology (99.5%) [27]. In a study published in 2017, Ferreira et al. described both vaccines and how they elicit long-lasting cellular and humoral responses. For innate immunity, the 17D-204 vaccine mobilizes myeloid and plasmacytoid dendritic cells, which are crucial for initiating the adaptive immune response. The vaccine promotes a rapid and intense proliferation of CD8+ T lymphocytes, followed by their transition to a memory cell phenotype, which is responsible for long-term immunity against YFV. The 17D-204 vaccine also initially reduces the number of B lymphocytes, reflecting the activation of the humoral response and the production of neutralizing antibodies capable of providing protection against YFV. It also significantly increases the production of chemokines IP-10 and IL-1α, which are associated with the antiviral response of the innate immune system. Thus, the variation in cytokine production reflects different patterns of activation and regulation of the inflammatory response in vaccinated individuals [28].

In contrast, the 17DD vaccine induces an innate response characterized by the activation of neutrophils and increased expression of markers, such as CD28 and HLA-DR, in different cell subtypes including eosinophils and monocytes. The 17DD vaccine also activates CD4+ and CD8+ T lymphocytes, with CD4+ T cells displaying late activation markers that remain elevated up to 30 days post-vaccination, suggesting a more gradual, yet effective, immune response. This pattern of CD4+ T-cell activation is associated with a mixed response, featuring characteristics of both Th1 and Th2 responses, indicating modulation of the inflammatory response. Regarding B lymphocytes, the 17DD vaccine presents a balance between activation and modulation, with an increase in early activated B cells that supports their proliferation and maturation [28].

Thus, while the 17D-204 vaccine tends to promote a more intense and early innate response, the 17DD vaccine is associated with a more modulated and gradual inflammatory and adaptive response.

The data also revealed that a small portion of the population (around 12.4%) did not show neutralizing antibodies against the 17DD strain. This number was higher with respect to 17D-204 (22%). These findings raise questions about vaccine efficacy or potential variations in individual immune responses.

Critically, these results suggest that although the 17D-204 and 17DD strains are closely related, the immune responses they elicit are not identical. This might have implications for vaccine strategies, particularly in regions where one strain is predominantly used. Moreover, the higher efficacy of the 17DD strain in eliciting neutralizing antibodies could be a factor to consider when evaluating the overall protective capacity of yellow fever vaccination programs.

However, this study has limitations that warrant attention. The sample size, while considerable, may not fully capture the heterogeneity of immune responses in broader populations, especially in individuals exposed to multiple orthoflaviviruses. Notably, this study did not explore the longevity of these neutralizing responses, which would be favorable for understanding the long-term efficacy of the vaccines.

## 5. Conclusions

Our study reveals higher neutralizing antibody titers to homologous 17DD sub-lineage compared to heterologous 17D-204 in the general population. Despite their high genetic similarity, these strains exhibit distinct immunogenic responses. Additionally, the finding that a portion of the population showed no detectable antibodies to either strain raises questions about individual immune variability and the efficacy of these vaccines. These insights emphasize the need for further research into the specific immune mechanisms and longevity of responses induced by different yellow fever vaccines, which could have important implications for optimizing immunization strategies.

## Data Availability

The dataset from this study is available on the Mendeley Data platform and may be accessed through the following doi: 10.17632/xt5xhspfbf.2.

## References

[1] ICTV (2023). Virus Taxonomy: 2023 Release (MSL #39).

[2] Pierson T.C., Diamond M.S., Knipe D.M., Howley P.M. (2013). Flaviviruses. Fields Virology.

[3] Fischer C., de Oliveira-Filho E.F., Drexler J.F. (2020). Viral emergence and immune interplay in flavivirus vaccines. Lancet Infect. Dis..

[4] Ishikawa T., Yamanaka A., Konishi E. (2014). A review of successful flavivirus vaccines and the problems with those flaviviruses for which vaccines are not yet available. Vaccine.

[5] Dutta S.K., Langenburg T. (2023). A Perspective on Current Flavivirus Vaccine Development: A Brief Review. Viruses.

[6] Poland J.D., Calisher C.H., Monath T.P., Downs W.G., Murphy K. (1981). Persistence of neutralizing antibody 30–35 years after immunization with 17D yellow fever vaccine. Bull. World Health Organ..

[7] Ma J., Boudewijns R., Sanchez-Felipe L., Mishra N., Vercruysse T., Buh Kum D., Thibaut H.J., Neyts J., Dallmeier K. (2021). Comparing immunogenicity and protective efficacy of the yellow fever 17D vaccine in mice. Emerg. Microbes Infect..

[8] James E.A., LaFond R.E., Gates T.J., Mai D.T., Malhotra U., Kwok W.W. (2013). Yellow fever vaccination elicits broad functional CD4^+^ T cell responses that recognize structural and nonstructural proteins. J. Virol..

[9] Akondy R.S., Monson N.D., Miller J.D., Edupuganti S., Teuwen D., Wu H., Quyyumi F., Garg S., Altman J.D., Del Rio C. (2009). The yellow fever virus vaccine induces a broad and polyfunctional human memory CD8^+^ T cell response. J. Immunol..

[10] World Health Organization (2015). Vaccines and vaccination against yellow fever: WHO Position Paper, June 2013—Recommendations. Vaccine.

[11] Gardner C.L., Ryman K.D. (2010). Yellow fever: A reemerging threat. Clin. Lab. Med..

[12] Beck A.S., Barrett A.D. (2015). Current status and future prospects of yellow fever vaccines. Expert. Rev. Vaccines.

[13] Theiler M., Smith H.H. (1937). The effect of prolonged cultivation in vitro upon the pathogenicity of yellow fever virus. J. Exp. Med..

[14] Staples J.E., Monath T.P. (2008). Yellow Fever: 100 Years of Discovery. JAMA.

[15] Monath T.P., Vasconcelos P.F. (2015). Yellow fever. J. Clin. Virol..

[16] Barrett A.D., Teuwen D.E. (2009). Yellow fever vaccine—How does it work and why do rare cases of serious adverse events take place?. Curr. Opin. Immunol..

[17] Brasil Imunizações—Cobertura—Brasil. http://tabnet.datasus.gov.br/cgi/dhdat.exe?bd_pni/cpnibr.def.

[18] Vasilakis N., Durbin A.P., Travassos da Rosa A.P.A., Munoz-Jordan J.L., Tesh R.B., Weaver S.C. (2008). Antigenic Relationships between Sylvatic and Endemic Dengue Viruse. Am. J. Trop. Med. Hyg..

[19] Vasilakis N., Shell E.J., Fokam E.B., Mason P.W., Hanley K.A., Estes D.M., Weaver S.C. (2007). Potential of ancestral sylvatic dengue-2 viruses to re-emerge. Virology.

[20] Halstead S.B. (1988). Pathogenesis of dengue: Challenges to molecular biology. Science.

[21] Monath T.P. (2001). Yellow fever: An update. Lancet Infect. Dis..

[22] Halstead S.B. (2017). Dengvaxia sensitizes seronegatives to vaccine enhanced disease regardless of age. Vaccine.

[23] Saito Y., Moi M.L., Takeshita N., Lim C.K., Shiba H., Hosono K., Saijo M., Kurane I., Takasaki T. (2016). Japanese encephalitis vaccine-facilitated dengue virus infection-enhancement antibody in adults. BMC Infect. Dis..

[24] Camacho L.A., Freire Mda S., Leal Mda L., Aguiar S.G., Nascimento J.P., Iguchi T., Lozana Jde A., Farias R.H., Collaborative Group for the Study of Yellow Fever Vaccines (2004). Immunogenicity of WHO-17D and Brazilian 17DD yellow fever vaccines: A randomized trial. Rev. Saude Publica.

[25] de Melo A.B., da Silva Mda P., Magalhaes M.C., Gonzales Gil L.H., Freese de Carvalho E.M., Braga-Neto U.M., Bertani G.R., Marques E.T., Cordeiro M.T. (2011). Description of a prospective 17DD yellow fever vaccine cohort in Recife, Brazil. Am. J. Trop. Med. Hyg..

[26] Reinhardt B., Jaspert R., Niedrig M., Kostner C., L’Age-Stehr J. (1998). Development of viremia and humoral and cellular parameters of immune activation after vaccination with yellow fever virus strain 17D: A model of human flavivirus infection. J. Med. Virol..

[27] Jennings A.D., Whitby J.E., Minor P.D., Barrett A.D. (1993). Comparison of the nucleotide and deduced amino acid sequences of the structural protein genes of the yellow fever 17DD vaccine strain from Senegal with those of other yellow fever vaccine viruses. Vaccine.

[28] Ferreira C.C., Campi-Azevedo A.C., Peruhype-Magalhães V., Costa-Pereira C., Albuquerque C.P., Muniz L.F., de Souza T.Y., Oliveira A.C.V., Martins-Filho O.A., da Mota L.M.H. (2017). The 17D-204 and 17DD yellow fever vaccines: An overview of major similaritis and subtle differences. Expert. Rev. Vaccines.

